# Supplementary figures and images for: A machine learning approach to predict extreme inactivity in COPD patients using non-activity-related clinical data
Source: PLoS One. 2021 Aug 19;16(8):e0255977. doi: 10.1371/journal.pone.0255977 (PMC8376055; doi:10.1371/journal.pone.0255977)

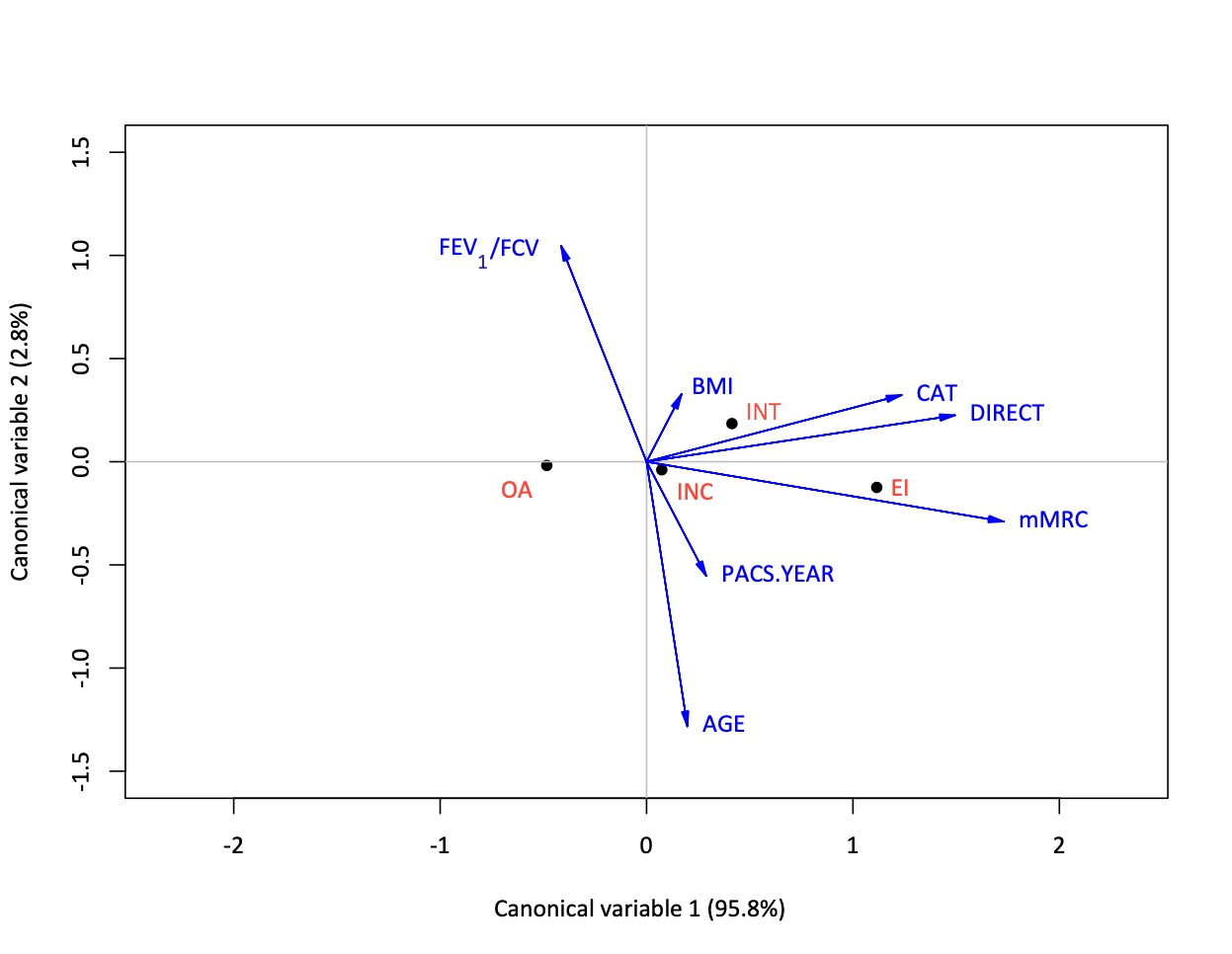

Supplement: S1 Fig — The two dimensions account for 98.6% of the variance between categories, most (95.8%) of which is due to EI versus OA. The latter is mainly influenced by FEV1/FVC and the former by CAT, DIRECT, and mMRC scores. (TIFF) [file pone.0255977.s001.tiff]

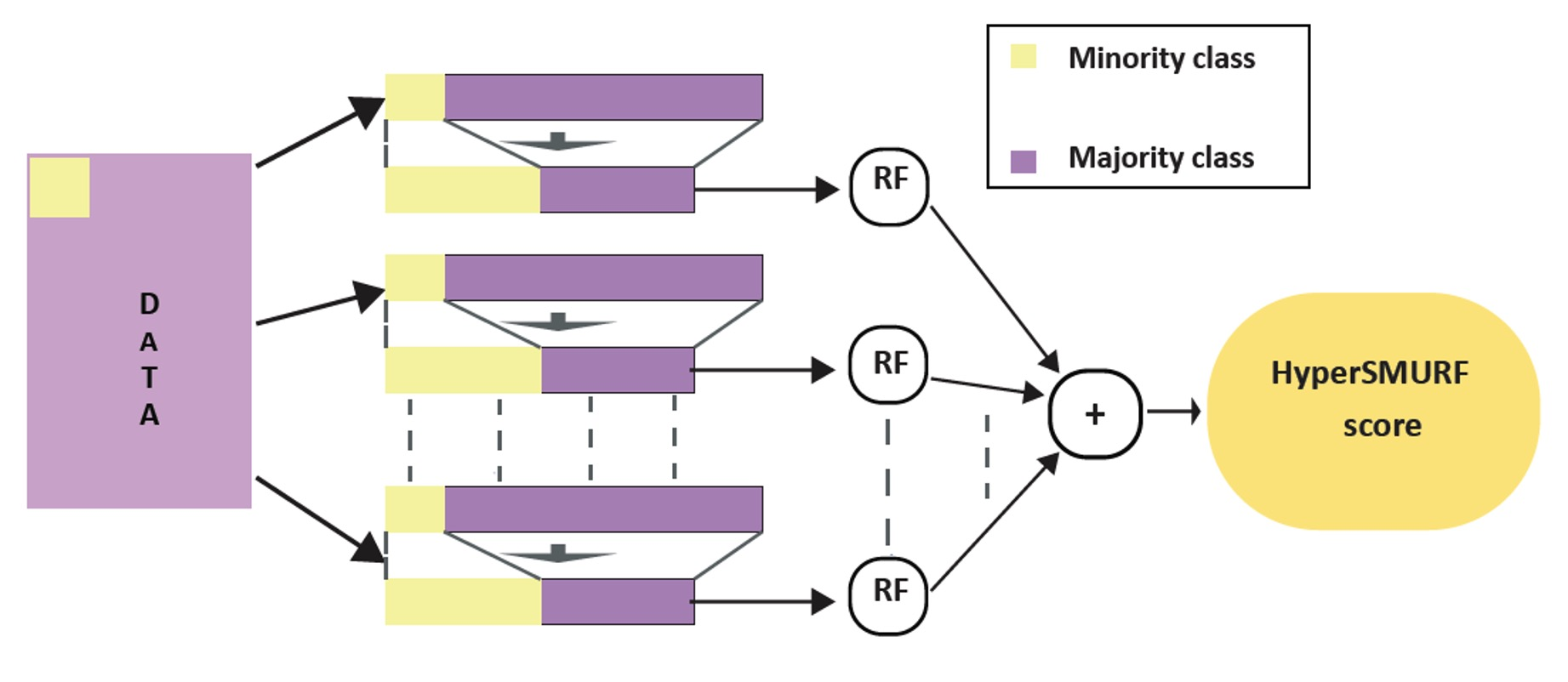

Supplement: S2 Fig — HyperSMURF divides the majority class (OA) into n partitions. For each partition, oversampling techniques are used to generate additional patients from the minority class (EI) that closely resemble the distribution of the actual class to amplify the number of training patients from the minority class. At the same time, a comparable number of patients is subsampled from the majority class. HyperSMURF then trains in parallel n random forests on the resulting balanced data sets and finally combines the prediction of the n ensembles according to a hyper-ensemble (ensemble of ensembles) approach. (TIFF) [file pone.0255977.s002.tiff]

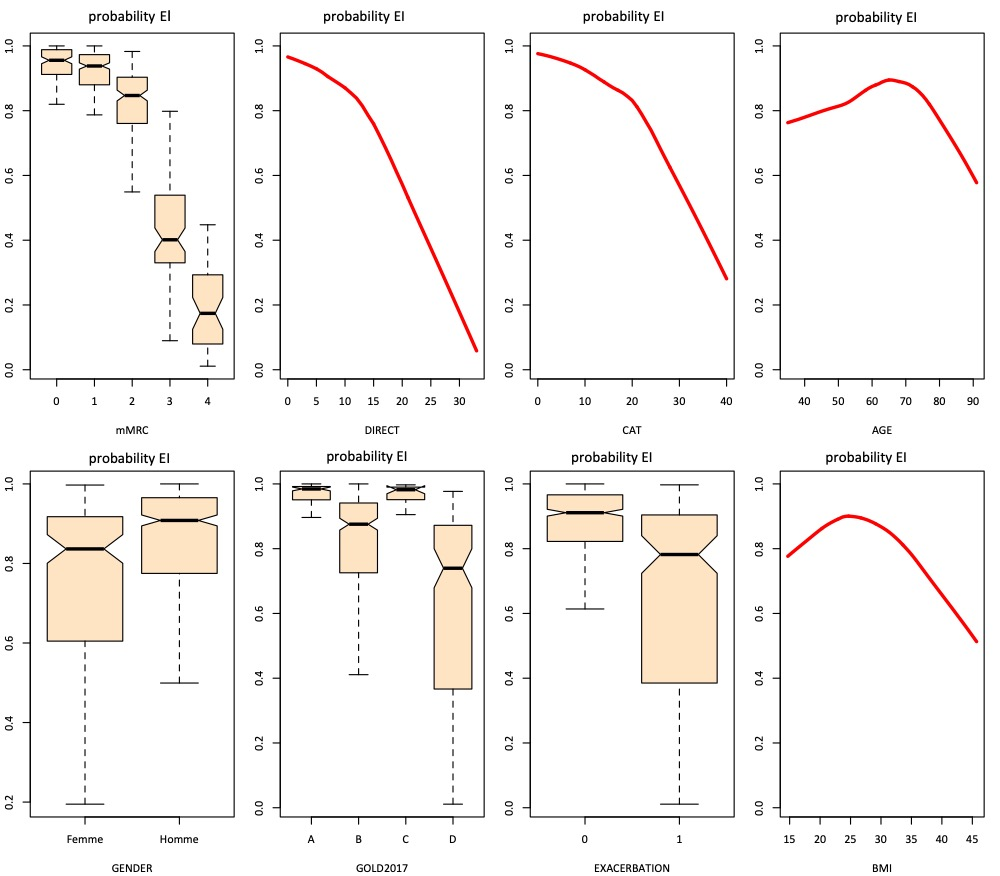

Supplement: S3 Fig — Box plots show the median, minimum, maximum, and interquartile values. See Table 1 for definitions of activity categories. (TIFF) [file pone.0255977.s003.tiff]
