# Supplementary material for: A machine learning approach to predict extreme inactivity in COPD patients using non-activity-related clinical data
Source: PLoS One. 2021 Aug 19;16(8):e0255977. doi: 10.1371/journal.pone.0255977 (PMC8376055; doi:10.1371/journal.pone.0255977)
Supplement: S1 Table — (PDF) [file pone.0255977.s004.pdf]

	INT				INC				
	Total (n=410)	a (n=226)	b (n=23)	c (n=161)	Total (n=167)	a (n=69)	b (n=37)	c (n=8)	d (n=53)
GOLD A	6.8%	6.2%	17.4%	6.2%	10.2%	5.8%	8.1%	37.5%	13.2%
GOLD B	60.5%	60.2%	47.8%	62.7%	50.3%	47.8%	45.9%	50.0%	56.6%
GOLD C	2.4%	0.4%	4.3%	5.0%	2.4%	0.0%	8.1%	0.0%	1.9%
GOLD D	30.2%	33.2%	30.4%	26.1%	37.1%	46.4%	37.8%	12.5%	28.3%

Data are presented as the percentage of patients within each category or subcategory. GOLD, Global Initiative for Chronic Obstructive Lung Disease. See table 1 for definitions of INT and INC categories and subcategories.
